# Costs of a school-based dental mobile service in South Africa

**DOI:** 10.1186/s12913-016-1827-2

**Published:** 2016-10-19

**Authors:** M. P. Molete, L. Chola, K. J. Hofman

**Affiliations:** 1 School of Oral Health Sciences, Faculty of Health Sciences, University of the Witwatersrand, Johannesburg, South Africa; 2 School of Public Health, Faculty of Health Sciences, University of the Witwatersrand, Johannesburg, South Africa; 3 Priority Cost-Effective Lessons for Systems Strengthening-South Africa (PRICELESS SA), University of Witwatersrand School of Public Health, Medical Research Council/Wits Rural Public Health and Health Transition Research Unit (Agincourt), Johannesburg, South Africa

**Keywords:** Mobile dental care, Oral health, Cost analysis, School based program

## Abstract

**Background:**

The burden of untreated tooth decay remains high and oral healthcare utilisation is low for the majority of children in South Africa. There is need for alternative methods of improving access to low cost oral healthcare. The mobile dental unit of the University of the Witwatersrand (Wits) has been operational for over 25 years, providing alternative oral healthcare to children and adults who otherwise would not have access. The aim of this study was to conduct a cost-analysis of a school based oral healthcare program in the Wits mobile dental unit. The objectives were to estimate the general costs of the school based program, costs of oral healthcare per patient and the economic implications of providing services at scale.

**Methods:**

In 2012, the Wits mobile dental unit embarked on a 5 month project to provide oral healthcare in four schools located around Johannesburg. Cost and service use data were retrospectively collected from the program records for the cost analysis, which was undertaken from a provider perspective. The costs considered included both financial and economic costs. Capital costs were annualised and discounted at 6 %. One way sensitivity tests were conducted for uncertain parameters.

**Results:**

The total economic costs were R813.701 (US$76,048). The cost of screening and treatment per patient were R331 (US$31) and R743 (US$69) respectively. Furthermore, fissure sealants cost the least out of the treatments provided. The sensitivity analysis indicated that the Wits mobile dental unit was cost efficient at 25 % allocation of staff time and that a Dental Therapy led service could save costs by 9.1 %.

**Conclusions:**

Expanding the services to a wider population of children and utilising Dental Therapists as key personnel could improve the efficiency of mobile dental healthcare provision.

## Background

The prevalence of untreated dental caries is 35 % globally and ranks in the top 100 major contributors of Disability Adjusted Life Years (DALYs), [1]. It is also one of the prevalent conditions experienced by school children in South Africa [2, 3]. Recent studies undertaken in Gauteng and Kwa Zulu Natal provinces showed that amongst 6–8 year olds, the caries prevalence was 46 and 73 % respectively, and of concern was that more than 90 % of the decay amongst the children went untreated [4, 5]. Untreated decay impacts negatively on families and children’s quality of life [6]. The effects to families arise due to disruption of life routines and absenteeism from work. In children, effects include pain, difficulty in eating, sleeping, increased hospital admissions and school absenteeism [7–9]. There is thus a need to introduce oral health care interventions early, in the primary years of children in order to delay the onset and control the severity of decay [10]. Furthermore, frequent and early dental visits amongst children may result in fewer curative visits and lower patient costs [8]. Despite these benefits of early treatment, access to oral healthcare remains low for the majority of children in South Africa. A study conducted in Limpopo province found that among 12–14 year olds (*n* = 1103), only 31.3 % of children had ever been to a dentist [11]. This is indicative of poor access to care and hence there is a need for alternative low cost and easily accessible oral health services for approximately 12 million children attending public schools in South Africa [12].

Mobile dental units have been used as alternatives to supplement the standard of care in order to reach underserved populations in many countries. They have been shown to be cost-efficient and highly successful in improving access [13–15]. A study comparing unit costs of fixed facility and mobile community based dental services in Thailand showed that the mobile intervention provided comprehensive oral health care at a lower cost [16]. In South Africa, studies have been undertaken to demonstrate the feasibility of providing primary health care in mobile dental units. Furthermore they have shown how preventative services such as fissure sealants undertaken in such facilities, can improve the oral health of school children [17–19]. The provision of fissure sealants with the use of mobile dental facilities is what the South African policy of re-engineering and strengthening of Primary Health Care aims to achieve [3]. According to a report from the Gauteng Oral Health Department, of the 10.4 million children aged 5–14 years in South Africa, only 0.5 % had benefitted from fissure sealant programmes [GDoH; Narrative Report on Oral Health to Province, unpublished].

The University of the Witwatersrand (Wits) operates a mobile dental service which is aligned to the PHC re-engineering policy in the country. It offers free oral health screening, preventative care and curative services at socially deprived schools in Johannesburg. Undertaking a cost analysis of these services was therefore necessary for gaining insight into the costs involved in the provision of oral health care with the use of mobile dental trucks to the many underserved school children in South Africa [3].

The aim of this study was to undertake a cost-analysis of a school based oral healthcare program. The objectives were to estimate the general costs of the program, costs of oral healthcare per patient and the implications of providing the services at scale.

## Methods

### Description of the programme

The Community Oral Health Outreach Project (COHOP) at Wits has been addressing the oral health needs of children for over 25 years with the use of a dental mobile truck. The services generally offered in the dental mobile unit include oral health screening, fissure sealants, fluoride applications, oral health education, simple extractions and restorations. Services that are beyond the scope of what the mobile unit offers are referred to the Wits Oral Health Centre at the Charlotte Maxeke Academic Hospital. The key personnel operating in the mobile unit include a Dentist, a Dental Therapist, a Dental Assistant and a Driver. When the need arises an Oral Hygienist is at times requested to assist in the programme.

### Target population

In the study, four government schools consisting of a total learner population of 2334 were visited for a period of 5 months in 2012 around the Hillbrow and Yeoville area in Johannesburg. The population included primary school children between the ages of 6 to 12 years old. The learners from the schools were from poor socio-economic backgrounds, some from child-headed families as parents had died or children were left with one sick parent who was unable to work in order to sustain the family. Before visiting the schools, COHOP sent screening consent forms to parents and caregivers via the school principals.

Out of a total of 2334 children, only 946 (41 %) consented to screening. Upon receiving written consent from parents or guardians, the schools were visited and oral health screenings were conducted. On completion of the screenings, parents or guardians were notified and additional consent was requested before treatment began. Children requiring extensive dental treatment or those that experienced extreme dental phobia were referred to the Wits Oral Health Centre.

### Description of the mobile dental truck

The vehicle housing the mobile equipment is a four tonne truck, fitted with two water tanks for fresh and contaminated water. Inside the truck, are two foot controlled dental chairs separated by a steel cabinet, each chair with a built-in operating light. The chairs are connected to dental units which consist of mountings for a three in one syringe, high speed and slow speed hand pieces, saliva ejector and high volume suction. There are also two operator and two assistants’ chairs in the unit.

### Costing

Costing was done from a public purchaser’s perspective, which in this context was the Gauteng Department of Health as they are the funders of primary health care services. Thus, only costs of managing the mobile unit were included in this analysis, and patient level costs of accessing the facility were not included. Data collection was guided by standard guidelines for costing health interventions [20–22]. A costing sheet containing all items to be included in the analysis was developed in Microsoft Excel, for the purposes of data collection. The primary source of data was the records of the mobile dental unit, which included billing records, patient records and financial accounts for the 2012 financial year. Data collected from the records included salaries, equipment, materials and supplies. The gross salaries of staff used were obtained from the salary scales provided by the Department of Health. Prices of materials and supplies were reflected in the financial records of the mobile dental unit. Costs were adjusted to 2012 prices using a Consumer Price Index (CPI) [23]. Prices were converted from the local currency (South African Rand - ZAR) to United States Dollars (US$) at an exchange rate of ZAR10.7 to US$1 (2012 average) [24].

The costing involved both financial and economic costs. Financial costs represented the expenditures used to purchase resources and operate the program and economic costs included the opportunity costs that reflect the value of the alternate use of resources [25].

Costs were further divided into capital and recurrent costs. Capital costs included costs lasting for more than 1 year such as the truck and dental equipment. Recurrent costs were costs that could be replaced within a year such as dental materials and supplies [20, 22]. A physical count of all capital items was undertaken, and only functional items were included in the analysis. Capital costs were annuitized in order to reflect their annual value. The annual financial cost of capital items was calculated using a straight line depreciation method, where the cost of an item is divided by its useful life years [20, 22]. The annual economic cost of capital items was calculated using a discount rate of 6 %, as recommended in the literature, and because it was close to the South African lending rate [26]. The useful life years were 11 years for the truck, 7 years for the vehicle and 3 years for the dental equipment [27].

Joint costs were considered in order to ensure that only costs attributed to the school program were taken into account [21]. The joint costs were those shared among four programs which were; oral health programs of the elderly, crèches, and individuals with physical and mental disabilities. Costs were allocated equally amongst the programs as there was no knowledge of the percentage allocation, 25 % of time was therefore allocated to each program and assumptions were tested in the sensitivity analysis.

### Outputs and average costs

Outputs assessed included the number of patients screened and those that had treatments involving extractions, conventional restorations, atraumatic restorative restorations (ART), fissure sealants and fluoride treatments. The impact of the intervention was determined by calculating the number of learners screened, treated and type of procedures offered. The total costs were then divided by the outputs (numbers screened, treated and type of procedures offered) in order to obtain the average costs of operating the mobile dental unit.

### Sensitivity analysis

A sensitivity analysis was undertaken in order to account for uncertainty [26]. One way sensitivity analyses were conducted on two uncertain variables: shared costs and task shifting of personnel. The shared costs were for personnel used between the school program and other COHOP programs. It was estimated that personnel worked 25 % of the time on the school based program, and the rest was on the other three programs. In the sensitivity analysis, the impact of allocating 50 and 100 % of the time to the mobile unit was tested. In addition, the impact of task shifting, from using dentists to employing dental therapists, who have a much lower salary was assessed.

## Results

During the study period, 946 learners were screened, and out of those, 421 were treated in a 5 month period. The amount of learners screened and treated was divided by 20 weeks and this translated to screening 47.30 and treating 21.05 learners per week. Using these figures, learners screened and treated per week was multiplied by 52 weeks to obtain the potential numbers that could be reached in a year. We thus estimated that the dental mobile unit could screen about 2459 and treat 1094 children per annum.

### Services offered

Within 5 months of the program, 946 children were screened and 421 of those children were treated. The following procedures were undertaken; 95 of the children were given fluoride applications; 1677 teeth received fissure sealants, 850 teeth were restored and 182 were extracted.

### Total costs

Table 1 shows the total economic costs of all inputs which amounted to R813,701 (US$76,048). Recurrent costs contributed to 57.5 % of all costs and capital costs contributed to 42.6 %. Personnel costs (34.3 %) contributed the most to recurrent costs followed by dental materials (18.1 %). Vehicles and equipment each contributed equally (21.3 %) to total capital costs.

**Table 1:** Total economic costs of inputs

	R	US$	Percentage contribution
Recurrent costs			
Personnel costs	279,364	26,109	34.3 %
Transport costs	6286	588	0.8 %
Vehicle maintenance	3952	369	0.5 %
Equipment maintenance	29,014	2712	3.6 %
Supplies	1657	155	0.2 %
Dental materials	146,980	13,736	18.1 %
Total recurrent costs	467,253	43,669	57.5 %
Capital costs			
Vehicles	172,983	16,167	21.3 %
Equipment	173,465	16,212	21.3 %
Total capital costs	346,448	32,379	42.6 %
Total costs	813,701	76,048	100 %

*R* South African Rands, *US$* United States Dollars

### Average costs

Table 2 shows the costs per patient over the 5 month period in which the project was conducted, and the projected average per year. For the 5 months, the average costs were R860 (US$80) for screening and R1932 (US$180) for treatment. Increasing the number of patients screened over a year period reduced costs by 61 %. The resultant annual average costs were R331 (US$31) for screening and R743 (US$69) for treatment per child. In terms of costs for the various procedures undertaken, fissure sealants undertaken were found to cost the least R485 (US$ 45) and conventional restorations cost the most R23,932 (US$ 2236) in treatments performed; (Table 3).

**Table 2:** Average costs per learner

Procedures	No of learner/5 months	Costs per learner (5 months)	Estimated No of learners/year	Costs per learner per year
Screening	946	R858 (US$ 80)	2460	R331 (US$ 31)
Treatment	421	R1928 (US$ 180)	1095	R743 (US$ 69)

*R* South African Rands, *US$* United States Dollars

**Table 3:** Average costs of procedures

Type of procedures	Amount of procedures	Costs of undertaking the various procedures
ART restorations	816 teeth	R997 (US$ 93)
Fissure sealants	1677 teeth	R485 (US$ 45)
Conventional restorations	34 teeth	R23,932 (US$ 2236)
Extractions	182 teeth	R4470 (US$ 417)
Fluoride applications	95 patients	R8565 (US$ 800)

*R* South African Rands, *US$* United States Dollars

### Sensitivity analysis

Changing the allocation of time to 50 % increased total costs from R813,700 (US$76,047) to R1,093,064 (US$102,155) (Table 3). At 100 % time allocation, costs further increased by 51 %. In terms of task shifting to a dental therapist at 25 % allocation of time, total program costs reduced by 9.1 % from R813,700 (US$76,047) to R739,316 (US$69,094) see (Tables 4 and 5).

**Table 4:** Sensitivity analysis of shared costs

	Change in shared costs		
	25 %	50 %	100 %
Total costs	R813701 (US$76,047)	R1,093,064 (US$102,155)	R1,651,791 (US$154,372)
Cost/patient screened	R331 (US$ 31)	R444 (US$ 41)	R672 (US$ 63)
Cost/patient treated	R743 (US$ 69)	R999 (US$ 93)	R1509 (US$141)

*R* South African Rands, *US$* United States Dollars

**Table 5:** Task shifting to Dental Therapists (at 25 % allocation)

Total costs	R739,316 (USD 69,094)
Cost/patient screened	R301 (US$ 28)
Cost/patient treated	R675 (US$ 63)

*R* South African Rands, *US$* United States Dollar

## Discussion

This study provides the costs of a school based oral healthcare service using a mobile unit in South Africa. Results show that personnel costs are the highest cost drivers, followed by vehicles, equipment and dental materials. These findings are similar to results in the West Rand study, which also showed that personnel salaries accounted for most of the mobile dental service costs [19]. Comparisons with this study are, however, limited, since it was undertaken on a different target population ranging from children to adults of various ages, and the study was conducted more than 10 years ago.

In terms of the average costs per patient, the study shows that the low (*n* = 946) uptake of screening services in 5 months resulted in 61 % higher costs than if more (*n* = 2460) learners were to take up screening over a full year. Furthermore, the higher costs of conventional restorations and fluoride applications may have resulted from the low uptake of those procedures. Similarly in Thailand, a study estimated unit costs for dental service delivery in both a hospital setting and a mobile dental setting. The authors found that while services on a mobile setting resulted in lower costs, the low uptake of certain procedures such as scaling in the unit resulted in high cost of the procedure [16].

The sensitivity analysis indicated that for COHOP, an increase in time allocation from 25 to 50 % and 100 %, increased the total costs by 34 and 51 % respectively. The analysis shows the potential costs if the mobile unit was operated at full capacity. While these total costs appear to be large at full scale, the average costs might actually be lower, considering that operating at full capacity will enable the unit to reach a larger number of children.

In the program only 41 % had consent for oral health screening. Increasing the number of learners to be screened and ultimately treated, is often a challenge experienced in school based programs, as uptake of screening and other services in the mobile dental unit are dependent on parental consent. Similar sentiments were expressed in an evaluation of a mobile dental fissure sealant programme in Hammanskraal, South Africa. The authors attributed the low levels of uptake due to poor parental consent and absenteeism on days when the dental mobile was present at the school premises [18]. Similar challenges were experienced in the study conducted in Thailand [16]. This study found that low utilisation affected fixed costs (labor, transportation costs) in a negative way in that the costs were shared by fewer patients; resulting in higher unit costs [16].

In the present study, fissure sealants were found to cost less in terms of the procedures undertaken. Given that they are one of the priority prevention strategies recommended to reduce dental caries amongst school children in South Africa [18]. Scaling up the uptake of school mobile dental screening and fissure sealant programmes in the country could potentially result in a reducing dental caries in an efficient manner.

Lower uptake of mobile dental services by learners in the study may have been a result of poor co-operation of school teachers and parental awareness. Therefore increasing parental awareness and teacher co-operation regarding mobile dental services could potentially increase screening uptake and subsequently reduce unit costs. In order to optimise the usage of the service, more community engagement should be undertaken in order advertise the services, and sensitise parents and other members of the community on the importance of oral health.

The analysis also indicated that as shown by evidence [19, 28] it may be cost effective to have the program operated predominantly by Dental Therapists, who have sufficient skills to provide primary oral health services at a lower cost. On the other hand Dentists have additional skills which could be best utilised to provide services in secondary care settings. Though the cost saving from a Dentist to a Dental Therapist was only found to be 9.1 %, when scaling up to other districts or provinces, it may result in huge cost savings.

### Limitations

The time frame used in this study was limited, and in future, data should be collected over a longer period in order to reduce uncertainty and to capture some seasonal effects. Furthermore, due to its retrospective nature, the study was undertaken from a provider perspective, and did not take into account patient and societal costs, which would likely have demonstrated cost savings on travelling to and from fixed clinics, loss of productivity due to parents missing work and school absenteeism by learners. To determine a holistic view of the health benefits and costs of running a mobile dental unit for primary health care services, future research should look into a full economic evaluation comparing a mobile unit to a fixed clinic and in addition evaluate the different programs offered by the mobile dental unit.

## Conclusions

This study aimed to demonstrate the costs of providing oral health care for school children in a mobile dental unit. Results indicated that personnel costs of staff were the major cost drivers. Low patient outputs increased cost per patient by 61 % and the sensitivity analysis indicated that a Dental Therapist led mobile dental service saved costs by 9.1 %. The information generated in this study will be useful to planning for the expansion of service provision to a wider population, particularly in the South African government’s plans to integrate school health programmes in the primary healthcare re-engineering programme [3]. The costs provided in this analysis are still useful to understanding the cost structures and potential investments when the provision of oral health services in schools is considered.

## Abbreviations

COHOP: Community Oral Health Outreach Program
CPI: Consumer Price Index
NHI: National Health Insurance
PHC: Primary Health Care
PRICELESS: Priority Cost Effective Lessons for Systems Strengthening
R: South African Rand
US$: United States Dollar
WHO: World Health Organisation
WITS: University of the Witwatersrand

## References

[1] Marcenes W, Kassebaum NJ, Bernabé E, Flaxman A (2013). Global burden of oral conditions in 1990–2010. A systematic analysis. J Dent Res.

[2] Van Wyk PJ, Van Wyk C (2004). Oral health in South Africa. Int Dent J.

[3] Department of Health. National Health Insurance for South Africa, Towards Universal Health Coverage. 2015; Available in: https://www.health-e.org.za/wp-content/uploads/2015/12/National-Health-Insurance-for-South-Africa-White-Paper.pdf. Accessed 04 June 2016

[4] Thekiso M, Yengopal V, Rudolph MJ, Bhayat A (2012). Caries status among children in the West Rand District of Gauteng Province, South Africa. South African Dental Journal.

[5] Reddy M, Singh S (2015). Dental caries status in six year old children at Health Promoting Schools in KwaZulu Natal, South Africa. South African Dental Journal.

[6] Casamassimo PS, Thikkurissy S, Edelstein BL, Maiorini E (2009). Beyond the dmft: the human and economic cost of early childhood caries. J Am Dent Assoc.

[7] Bastos JL, Peres MA, Peres KG, Araujo CLP, Menezes AMB (2008). Toothache prevalence and associated factors: a life course study from birth to age 12 years. Eur J Oral Sci.

[8] Moles DR, Ashley P (2008). Hospital admissions for dental care in children. English 1997–2006. Br Dent J.

[9] Martins-Junior PA, Oliveira M, Marques LS, Ramos-Jorge ML (2012). Untreated dental caries: impact on quality of life of children of low socioeconomic status. Paediatric Dentistry.

[10] Sen B, Blackburn J, Morrisey MA, Kilgore ML (2013). Effectiveness of preventive dental visits in reducing non-preventative dental visits and expenditures. Pediatrics.

[11] Ayo-Yusuf OA, Okagbare TE, Ayo- Yusuf (2011). Prevalence and socio-economic disparities in fissure sealant placement among adolescents in the Limpopo Province, South Africa. South African Dental Journal.

[12] Department of Health & Basic Education. Integrated School Health Policy. 2012; Available in: https://www.health-e.org.za/2013/10/24/integrated-school-health-policy. Accessed 04 June 2016

[13] Rudolph MJ, Chikte UME, Lewis HA (1992). A mobile dental system in Southern Africa. J Public Health Dent.

[14] Clarke JR, Bradnock G, Hamburger R (1992). The uptake and completion of dental treatment using a mobile clinic in central Birmingham UK. Community Dent Health.

[15] Diaz-Perez Mde J, Farley T (2004). A program to improve access to health care among Mexican immigrants in rural Colorado. Journal of Rural Health.

[16] Tianviwat S, Chongsuvivatwong V, Birch S. Estimating Unit Costs for Service Delivery in Institutional and Community-Based Settings in Southern Thailand. 2008; Available in: http://aph.sagepub.com/content/early/2008/11/17/1010539508327246. Accessed 15 Oct 2015 10.1177/1010539508327246 19124339

[17] Kroon J, Prince E, Denicker GA (2001). Trends in treatment performed in the Phelophepa Dental Clinic: 1995–2000. South African Dental Journal.

[18] Van Wyk PJ, Kroon J, White JG (2004). Evaluation of a fissure sealant program as part of community-based teaching and training. J Dent Educ.

[19] Holtshousen WSJ, Smit A (2007). A cost-efficiency analysis of a mobile dental clinic in the public service. South African Dental Journal.

[20] World Health Organization. Unit financial costs. In: Creese A, Parker D, editors. Cost analysis in primary health care. Geneva; 1994. p. 5–51

[21] Drummond M, Sculpher M, Torrance G, O’Brien B, Stoddart G (2005). Methods for the economic evaluation of health care programmes.

[22] World Health Organization***.*** Estimating costs. In: Edejer TT, Baltussen R, Adam T, Hutubessy R. et al. editors. Guide to cost-effectiveness analysis. Geneva; 2003. p. 28–48

[23] Statistics South Africa. Consumer Price Index. 2012; Available in:http://www.statssa.gov.za/publications/P0141/CPIHistory.pdf. Accessed on 12/11/2015

[24] World Bank. World Development Indicators. 2014; Available in: https://data.worldbank.org/sites/default/files/wdi-2014-book.pdf. Accessed 02 June 2016

[25] Mc Pake B, Normand (2008). Issues in the measurement of costs. Health economics: an international perspective.

[26] South African Reserve Bank. Current Market Rates. 2015; Available in: https://www.resbank.co.za/Research/Rates/Pages/CurrentMarketRates.aspx; Accessed on 12/11/2015

[27] Magyorosy, Smith. The main methodological issues in costing health care services. 2005; Literature Review: Published by Centre for Health Economics

[28] Howard B, Beazoglou T, Drozdowski M (2008). Financial feasibility of a model school-based dental program in different States. Public Health Rep.

